# Influence of Polymers Diversity on the Dissolution Kinetics of Encapsulated *p*-Coumaric Acid in Oral Systems

**DOI:** 10.3390/gels11120983

**Published:** 2025-12-07

**Authors:** Monika Jokubaite, Vakare Jokubaityte-Tunkeviciene, Kristina Ramanauskiene

**Affiliations:** 1 Department of Drug Chemistry, Faculty of Pharmacy, Lithuanian University of Health Sciences, Sukileliai Avenue 13, LT-50162 Kaunas, Lithuania; 2 Department of Clinical Pharmacy, Faculty of Pharmacy, Lithuanian University of Health Sciences, Sukileliai Avenue 13, LT-50162 Kaunas, Lithuania; vakare.jokubaityte@stud.lsmu.lt (V.J.-T.); kristina.ramanauskiene@lsmu.lt (K.R.)

**Keywords:** *p*-coumaric acid, polymers, dissolution, antioxidant

## Abstract

*p*-coumaric acid is a natural phenolic compound with antioxidant activity, but its poor solubility and low bioavailability limit its practical use in oral formulations. The aim of this study is to evaluate how different polymers affect the dissolution and antioxidant properties of *p*-coumaric acid when incorporated into capsules and gels. Nine capsule compositions were prepared using poloxamer 407 (C1 group), sodium carboxymethyl cellulose (C2 group), chitosan (C3 group) and 5 hydrogels (G group) with the same polymers. The results indicate that *p*-coumaric acid is poorly soluble in aqueous solvents. The selected polymers modified the solubility of *p*-coumaric acid. The best solubility of *p*-coumaric acid was observed in mixtures containing 14.3% Poloxamer 407 (P407). The results showed that the polymers significantly affected the release kinetics of *p*-coumaric acid from the modeled capsules and gels. It was observed that capsules containing 14.3% P407 and gels with 14% P407 exhibited the best dissolution kinetics of *p*-coumaric acid. Antioxidant activity was strongest in formulations that released more *p*-coumaric acid. The results of this study confirm that the type and amount of excipients significantly affect the quality of capsules and gels. *p*-coumaric acid has the potential to be used in food supplements as a natural antioxidant, but further research is needed to improve its bioavailability and ensure safety.

## 1. Introduction

In recent years, increasing attention has been given to biologically active compounds due to their antimicrobial, anti-inflammatory and antioxidant properties. Modeling oral pharmaceutical forms, the application of excipients and their possible influence on the solubility and bioavailability of active substances is unavoidable. Many of these active compounds often exhibit poor solubility, which limits their application in the development of oral dosage forms. Researchers are extensively exploring the potential use of *p*-coumaric acid in oral dosage form formulations. *p*-coumaric acid is a phenolic acid, a secondary metabolite, hydroxyl derivative of cinnamic acid [1]. *p*-coumaric acid is widely distributed in various plants like apples, blueberries, pineapples, cereal grains, cinnamon and others [1]. Hydroxycinnamic acid derivatives, especially *p*-coumaric acid, exhibit antioxidant, anti-inflammatory, anticancer, antiulcer and antiplatelet effects [2]. These derivatives can reduce atherosclerosis, oxidative damage to the heart and nerves, UV-induced damage to eye tissue, anxiety. Isolated forms of hydroxycinnamic acid compounds have attracted more and more interest in the pharmaceutical field, as in vitro and in vivo studies have shown their antibacterial [3], antihyperlipidemic [4], immunomodulatory [5], neuroprotective [6,7], photoprotective [8] and other biological effects [9]. However, their efficacy by oral administration depends on bioavailability in the body [1,2]. A study conducted by Kim et al. described the pharmacokinetic properties of orally administered *p*-coumaric acid in humans [10]. The highest plasma concentration of *p*-coumaric acid was observed approximately 30 min after administration, with a half-life of about 55 min [10,11]. Another study demonstrated that the majority of *p*-coumaric acid is excreted via urine (54.1 ± 5.2% of the administered dose). The most intensive excretion of *p*-coumaric acid occurs within 0 to 6 h post-administration, indicating that the compound is rapidly absorbed, metabolized and eliminated from the body [11]. Zang et al. reported that *p*-coumaric acid exerts potent antioxidant activity through direct hydroxyl radical scavenging, leading to significant inhibition of lipid peroxidation and reduction in circulating LDL cholesterol in vivo, in this way indicating potential antiatherogenic properties [12].

For the potential application of *p*-coumaric acid, it is important to consider its biopharmaceutical properties. According to scientific data, *p*-coumaric acid has poor solubility in aqueous solvents and exhibits low bioavailability [13]. Poor solubility of active components is a major obstacle in the development of pharmaceutical formulations, as it limits the bioavailability of the active substance and causes problems due to its insufficient absorption. To address the solubility issue, approaches such as the formation of dispersion systems and the use of co-solvents are necessary. However, there is limited scientific data on the modeling of oral dosage forms containing *p*-coumaric acid, as research has primarily focused on the biological activity of this compound. Limited studies have been published on the influence of excipients on the dissolution kinetics of *p*-coumaric acid from oral dosage formulations. Solid dosage forms are among the most commonly used oral dosage forms due to their simple manufacturing process and convenient administration. In any oral dosage formulation, along with the active ingredient, excipients are used, which may influence the efficacy of the drug product [14]. Polymeric materials are extensively used in pharmaceutical formulations to modulate the release kinetics of active substances. It is essential to perform a biopharmaceutical evaluation of solid dosage form, including disintegration time and dissolution kinetics of the active substance. In this study, attention was focused on evaluating the influence of polymeric materials on the kinetics of *p*-coumaric acid released from capsules and edible hydrophilic gels. Hard capsules are among the most widely used solid oral pharmaceutical forms, whereas edible gels represent a promising alternative for individuals with swallowing difficulties, increasingly attracting the interest of researchers. Hydrogels were chosen as one of the most widely and increasingly studied drug delivery systems [15,16,17]. Literature data suggest that excipients can improve the solubility of active ingredients and modify the dissolution kinetics profile [14]. Cellulose derivatives, such as microcrystalline cellulose, are among the most commonly used excipients due to their favorable flow properties and compatibility with a wide range of active ingredients. Sodium carboxymethyl cellulose (NaCMC) is a widely used polysaccharide known for its excellent film-forming ability, water solubility, low cost and biodegradability. Limited studies have investigated the effect of P407 on the release of *p*-coumaric acid from oral pharmaceutical formulations [18,19]. Poloxamers belong to the group of nonionic block copolymers consisting of PEO (polyethylene oxide) and PPO (polypropylene oxide) units -PEO-PPO-PEO-. P407 is used as a surfactant, solubilizer, stabilizer, and lubricant, and it has the ability to form gel layers. Poloxamers enhance drug solubility and dissolution rate by reducing surface tension and forming micelles that promote the solubilization of active compounds [20]. Chitosan is a biocompatible, mucoadhesive polymer that forms drug-loaded nanoparticles in aqueous media [21,22,23]. However, its use in oral delivery is limited by its solubility at acidic pH, which diminishes its functional properties [21]. Most studies on p-coumaric acid have focused on its biological activity, whereas a systematic biopharmaceutical evaluation of its oral dosage forms is lacking. Comprehensive data are not available on how different polymeric excipients modulate the release and antioxidant performance of *p*-coumaric acid when incorporated into solid and semi-solid oral formulations. The novelty of this work lies in the parallel modeling of two patient-friendly antioxidant oral dosage forms—hard capsules and edible hydrophilic gels containing *p*-coumaric acid, combined with a comparative assessment of functionally distinct polymers. It is particularly relevant to characterize the dissolution kinetics of *p*-coumaric acid in these formulations and to integrate them with in vitro antioxidant activity in order to evaluate their biopharmaceutical performance. This approach provides new insights into the interactions between *p*-coumaric acid and selected polymers and offers a research platform for the future design of oral formulations containing poorly soluble phenolic acids. This study contributes to addressing a recognized key challenge in drug development—improving the solubility of the active substance and increasing its dissolution rate, in this way enhancing bioavailability. It is particularly important to evaluate the interactions of *p*-coumaric acid with selected polymeric materials and to assess their influence on solubility and dissolution kinetics when incorporated into different pharmaceutical formulations. The aim of this study is to model antioxidant oral dosage formulations using *p*-coumaric acid as a model active compound, and to evaluate how different polymeric excipients and dosage form affect their dissolution kinetics and antioxidant properties.

## 2. Results and Discussion

### 2.1. Solubility of p-Coumaric Acid

**Figure 1:**
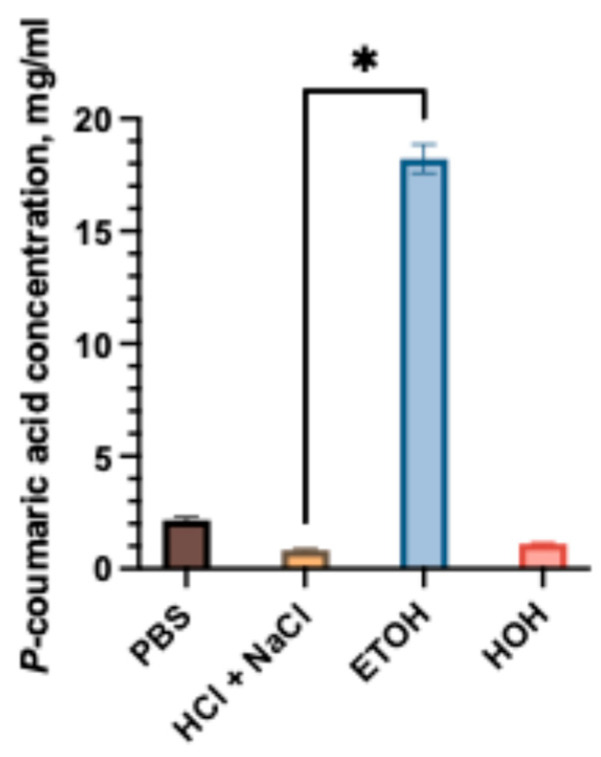
Solubility of *p*-coumaric acid in different media: PBS, water, HCl + NaCl acidic solution, and 96% ethanol. The asterisk (*) above the bars indicate statistically significant differences between groups (one-way ANOVA followed by Tukey’s multiple comparison test, *p* < 0.05, mean ± SD, *n* = 3).

One of the most important stages in pharmaceutical product development is the selection of an appropriate dosage form [24]. The selected polymers (poloxamer 407, NaCMC and chitosan) are widely used as excipients in oral dosage forms such as capsules and edible gels. Poloxamer 407 is typically incorporated at concentrations of about 15–25% (*w*/*w*) in thermoresponsive semi-solid formulations; within this range, it forms self-supporting gels, whereas at lower concentrations it produces less viscous, liquid-like systems [25]. The concentration range used for NaCMC hydrogels depends strongly on the molecular weight of the polymer, as higher molecular weight CMC forms mechanically stronger, more highly swollen networks than lower molecular weight analogs at comparable compositions [26,27]. Sodium carboxymethyl cellulose is usually employed at relatively high concentrations (approximately 5–10% *w*/*w*) to obtain self-standing hydrogels with adequate mechanical strength and swelling capacity, and several studies have reported optimal gel properties for 7% (*w*/*v*) NaCMC stock gels or matrices. In the present study, NaCMC concentrations of 7% and 14% (*w*/*w*) were selected to generate semi-solid systems and to approximate the polymer content used in the capsule formulations. Chitosan hydrogels are most often formulated at 1–5% (*w*/*w*) [28,29]. In our study, a 5% (*w*/*w*) chitosan gel was prepared to obtain a highly mucoadhesive yet still spreadable semi-solid whose polymer concentration is comparable to that in the capsule compositions. In this study, hard gelatin capsules and hydrogels were chosen as the delivery systems. The solubility of active compounds is one of the critical factors influencing the bioavailability of drugs; therefore, in the first stage of the study, the solubility of *p*-coumaric acid in different solvents was evaluated. The results of the study are presented in Figure 1.

The solubility of *p*-coumaric acid was evaluated in different media, including phosphate-buffered saline (PBS), hydrochloric acid with sodium chloride (pH 1.2), distilled water, and ethanol (96%). As shown in Figure 1, the solubility was strongly dependent on the solvent. The highest solubility was observed in ethanol, reaching 18.21 ± 0.64 mg/mL, while lower values were obtained in aqueous media. Among the aqueous systems, PBS exhibited the highest solubility (2.15 ± 0.13 mg/mL), which was statistically significantly greater (*p* < 0.05) compared to hydrochloric acid medium (0.82 ± 0.08 mg/mL). The results indicate that *p*-coumaric acid has poor aqueous solubility, with pronounced differences between neutral and acidic conditions. Our study results confirmed that *p*-coumaric acid is poorly soluble in aqueous solvents [13]. Considering the limited solubility in simulated gastrointestinal fluids, the selection of suitable excipients was essential for the development of *p*-coumaric acid capsule formulations. The compositions of the mixtures of *p*-coumaric acid and excipients are given in Table 2a.

**Figure 2:**
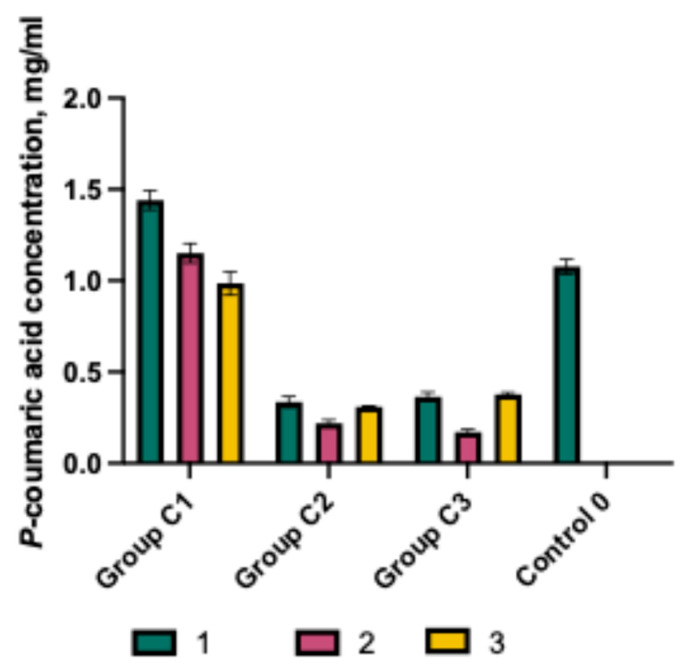
*p*-Coumaric acid concentration in purified water with various excipients and their combinations (mean ± SD, n = 3).

The effect of the selected capsule excipients on the solubility of p-coumaric acid was evaluated (Figure 2). The best solubility was observed in the C1 formulations, in which the predominant polymer was poloxamer 407. The solubility of *p*-coumaric acid in C1 formulations containing P407 was significantly higher than in groups C2 and C3. The highest dissolved amount was detected in capsules containing 100 mg of *p*-coumaric acid, 25 mg of P407, and 50 mg of Prosolv (C1-1), which was significantly greater compared to the control C0 (*p* < 0.05). Increasing the proportion of poloxamer 407 in formulations (C1-2, C1-3) resulted in decreased solubility of *p*-coumaric acid. The amount dissolved from the C1-2 C1-3 formulation was comparable to that of the control capsules C0. Overall, all C1 group mixtures showed significantly higher solubility of *p*-coumaric acid compared to capsule mixtures from groups C2 and C3. A review of the scientific literature confirms that poloxamer possesses solubility-enhancing properties for active ingredients and can influence the dissolution kinetics of poorly soluble substances [17]. These results also confirmed that the selected polymers differently modulate the solubility of *p*-coumaric acid. The inclusion of sodium carboxymethylcellulose and chitosan in the mixtures (C2 and C3) reduced the solubility of *p*-coumaric acid. The results of the studies showed that NaCMC and chitosan do not have a solubility-enhancing effect on *p*-coumaric acid, but rather form prolonged-acting gelled systems. It can be anticipated that the presence of different types of excipients in solid oral dosage forms may influence product quality, drug performance, and bioavailability. This is confirmed by scientific literature that the biopharmaceutical properties of product formulations also depend on the amount of selected excipients [30].

The effect of the selected capsule excipients on the solubility of p-coumaric acid was evaluated (Figure 2). The best solubility was observed in the C1 formulations, in which the predominant polymer was poloxamer 407. The solubility of *p*-coumaric acid in C1 formulations containing P407 was significantly higher than in groups C2 and C3. The highest dissolved amount was detected in capsules containing 100 mg of *p*-coumaric acid, 25 mg of P407, and 50 mg of Prosolv (C1-1), which was significantly greater compared to the control C0 (*p* < 0.05). Increasing the proportion of poloxamer 407 in formulations (C1-2, C1-3) resulted in decreased solubility of *p*-coumaric acid. The amount dissolved from the C1-2 C1-3 formulation was comparable to that of the control capsules C0. Overall, all C1 group mixtures showed significantly higher solubility of *p*-coumaric acid compared to capsule mixtures from groups C2 and C3. A review of the scientific literature confirms that poloxamer possesses solubility-enhancing properties for active ingredients and can influence the dissolution kinetics of poorly soluble substances [17]. These results also confirmed that the selected polymers differently modulate the solubility of *p*-coumaric acid. The inclusion of sodium carboxymethylcellulose and chitosan in the mixtures (C2 and C3) reduced the solubility of *p*-coumaric acid. The results of the studies showed that NaCMC and chitosan do not have a solubility-enhancing effect on *p*-coumaric acid, but rather form prolonged-acting gelled systems. It can be anticipated that the presence of different types of excipients in solid oral dosage forms may influence product quality, drug performance, and bioavailability. This is confirmed by scientific literature that the biopharmaceutical properties of product formulations also depend on the amount of selected excipients [30].

### 2.2. Capsules and Gels Quality Control

Quality control of capsules and gels involves various tests to ensure safety and efficacy during both product development and industrial manufacturing. In this study, the main capsule quality tests focused on mass uniformity, disintegration, and dissolution kinetics parameters. For the modeled gels, quality control prioritized pH value, viscosity, and active substance release tests, as well as base evaluation. The biological activity of capsules and gels containing *p*-coumaric acid was assessed by performing an antioxidant activity assay.

#### 2.2.1. Disintegration Test of Capsules

The disintegration process of capsules is a critical stage that ensures the bioavailability of the active substance from the pharmaceutical dosage form.

**Figure 3:**
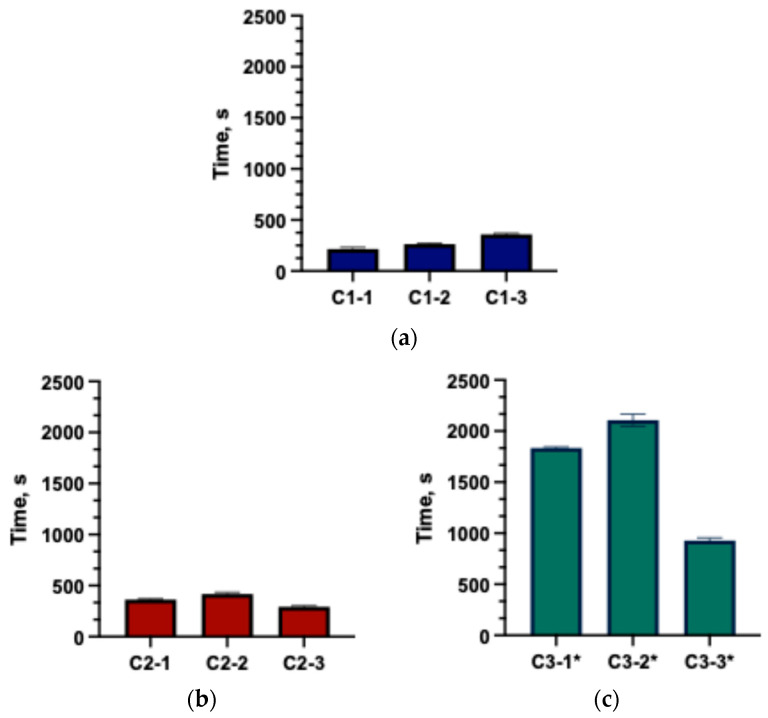
Disintegration times of the formulated capsules (mean ± SD, n = 3). (**a**) C1 group; (**b**) C2 group; (**c**) C3* group (* the capsule did not fully disintegrate; only the disintegration onset time was recorded).

The disintegration test is a mandatory procedure in the quality evaluation of capsules. The results (Figure 3) demonstrated that excipients had a significant impact on capsule disintegration time. The fastest disintegration was observed in capsules from groups C1 and C2. Capsules in group C3-1 and C3-2 demonstrated prolonged disintegration, with no complete disintegration observed within 30 min. Literature data indicate that low concentrations of chitosan (approximately 8.2%) can accelerate the disintegration of meloxicam tablets [21,31], whereas Binti Sarun et al. describe that higher concentrations (14%) prolong the disintegration of amoxicillin tablets due to hydrogel layer formation [32]. The findings confirmed that capsules disintegration time is determined by both the type and the concentration of excipients incorporated into solid formulations. Soraya et al.’s results show that PVP and NaCMC markedly increase water uptake and swelling of the matrices, which in turn promotes more efficient disintegration through a swelling-controlled mechanism. Upon hydration, dissociation of Na^+^ from the NaCMC backbone increases the number of ionized carboxymethyl groups, enhances intra- and intermolecular electrostatic repulsion and opens the polymer network, thereby amplifying its ability to swell. Such behavior is advantageous in dosage forms designed for controlled or sustained release [33]. In the study by Noreen et al. HA/Poloxamer 407 hydrogels exhibited dense porous microstructure and strong physical interactions between hyaluronic acid and Poloxamer 407 micelles, which accounted for the considerable swelling observed during the first hours of dissolution and the slower dissolution of these systems [34]. Chitosan has been extensively studied as a multifunctional excipient in solid oral dosage forms. Depending on the formulation, it can act as a disintegrant, a matrix-forming polymer in modified-release systems, or a solubility and permeability-enhancing agent. Its mucoadhesive and swellable nature contributes to improved dissolution [35].

#### 2.2.2. Physicochemical Properties of Gels

One of the main advantages of oral gels is that incorporation of the active ingredient into polymer matrix eliminates the need for a disintegration stage. Nevertheless, it is essential to evaluate their physicochemical properties, particularly pH and viscosity (Table 1). Viscosity is regarded as a critical quality parameter because it directly affects spreadability, residence time on the application site, and the release profile of the active substance, which in turn influences therapeutic performance [36,37]. The pH of the formulation plays a dual role; it ensures the chemical stability of the incorporated compound and contributes to patient acceptability by maintaining compatibility with the site of administration.

The physicochemical properties of the formulated gels depend on both the choice of gelling agent and its concentration. In this study, gel pH values ranged from 3.71 to 5.81, with the lowest value observed in the chitosan-based gel. According to recent literature, acidic formulations (low pH) can disrupt the oral cavity’s pH balance, contributing to dental erosion (Alves et al., 2025) [38]. Other tested gels exhibited higher pH values, suggesting a potentially safer profile compared to chitosan-based formulations. The type of gelling agent significantly influenced gel viscosity. Among all formulations, the 14% poloxamer gel exhibited the lowest viscosity. Poloxamer 407-based gels display thermoresponsive behavior, with viscosity substantially increasing as temperature rises [39]. In contrast, other gels showed decreased viscosity at higher temperatures.

### 2.3. Dissolution Test of Capsules and Gels Containing p-Coumaric Acid

**Figure 4:**
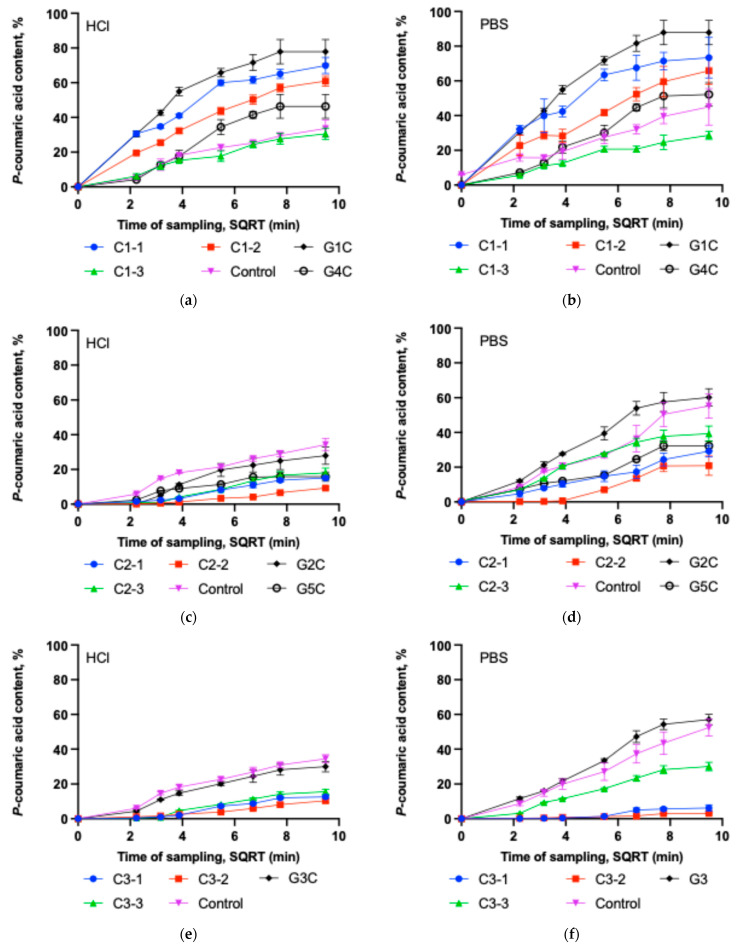
Dissolution test of *p*-coumaric acid versus the square root of time (SQRT) in 0.1 M hydrochloric acid and PBS (pH = 6.8) solutions for different capsule formulations and gels (mean ± SD, *n* = 3). (**a**) Dissolution of C1-1, C1-2, C1-3, control capsules and G1C and G4C gels in 0.1 M HCl. (**b**) Dissolution of C1-1, C1-2, C1-3, control capsules and G1C and G4C gels in PBS. (**c**) Dissolution of C2-1, C2-2, C2-3, control capsules and G2C and G5C gels in 0.1 M HCl. (**d**) Dissolution of C2-1, C2-2, C2-3, control capsules, and G2C and G5C gels in PBS. (**e**) Dissolution of C3-1, C3-2, C3-3, control capsules and G3C gel in 0.1 M HCl. (**f**) Dissolution of C3-1, C3-2, C3-3, control capsules and G3C gel in PBS.

Dissolution testing is a mandatory procedure for solid oral dosage forms and is routinely applied throughout product development to evaluate drug release and stability under simulated gastrointestinal conditions [40]. This test is also performed for oral suspensions; in our study, it was applied to gels in which *p*-coumaric acid was incorporated in a suspension form. The dissolution behavior of *p*-coumaric acid was investigated in both acidic (0.1 M HCl, pH 1.2) and buffered (PBS, pH 6.8) media. The results demonstrated that excipients significantly influenced the release kinetics of *p*-coumaric acid. The dissolution test results for capsules and gels are presented in Figure 4.

It was observed that C1-1 and C1-2 capsules exhibited better dissolution kinetics of *p*-coumaric acid compared to the other tested capsule groups. Capsule formulations C1-1, C1-2 and C1-3 in HCl solution released, respectively, 69.88%, 60.98%, and 30.54% of p-coumaric acid within 90 min (Figure 4a). After 90 min, the amount of *p*-coumaric acid released into the PBS medium by capsule formulations C1-1, C1-2 and C1-3 remained similar at 73.34%, 65.88%, and 28.68%, respectively (Figure 4b). The results of the study indicate that increasing the amount of Poloxamer leads to a slower dissolution of the active substance from the tested capsules and gels. The highest amount of the active substance was released from the Poloxamer-based gel G1C in both acceptor media (HCl and PBS), compared to gel G4C and all capsule formulations of the C1 group. It should be noted that Poloxamer affects the dissolution kinetics of *p*-coumaric acid differently in solid and gel forms. Although the capsules C1-1 and gel G1 contained similar amounts of gelling agent (14.3% and 14%, respectively), the release of *p*-coumaric acid from gel G1 was more intensive compared to the C1-1 capsules. Incorporating the active substance directly into the gel form avoids poloxamer dissolution and gelation processes, which may have resulted in more efficient *p*-coumaric acid dissolution kinetics compared to the capsules. During this study, it was also observed that the 25% Poloxamer gel G4 released a significantly lower amount of active substance after 90 min compared to the C1-2 capsules, which also contained 25% Poloxamer (P407). In contrast, the relatively high amount of poloxamer used in the G4C formulation could have led to slower *p*-coumaric acid release due to the formation of fewer hydrated micelles and temperature-induced aggregated structures such a gels [41,42]. Capsules containing sodium carboxymethyl cellulose (group C2) in Figure 4c,d demonstrated slow dissolution kinetics. Additionally, group C2 capsules exhibited lower *p*-coumaric acid release in acidic medium compared to the PBS medium, likely due to interactions between NaCMC and the acid, forming internal crosslinks (croscarmellose formation) that reduce solubility. Capsule formulations C2-1, C2-2 and C2-3 in HCl solution released, respectively, 15.14%, 9.30%, and 18.01% of *p*-coumaric acid within 90 min. After 90 min, the amount of *p*-coumaric acid released into the PBS medium by capsule formulations C2-1, C2-2 and C2-3 remained at 29.24%, 20.89%, and 39.31%, respectively. In the gels prepared with sodium carboxymethyl cellulose as the base, the release of *p*-coumaric acid depended on the amount of gelling agent. Gel G5 exhibited a statistically significantly higher viscosity compared to gel G1, which may have resulted in slower dissolution kinetics due to less efficient release of *p*-coumaric acid from the gel matrix. A significantly lower amount of gelling agent (7%) enabled the formation of a high-quality semi-solid gel (G2C), from which a statistically significantly higher amount of *p*-coumaric acid dissolved in the tested acceptor media after 90 min compared to gel G5C and C2 group capsules. Sodium carboxymethyl cellulose, due to its binding and viscosity-regulating properties, allows controlled drug release [43]. In this study, the selected concentration of sodium carboxymethyl cellulose (14–40%) prolonged the dissolution kinetics of *p*-coumaric acid. Published research results indicate that the use of CMC in powder form can enhance nasal drug absorption due to its prolonged-release effect [43,44]. Our study demonstrated that this water-soluble cellulose derivative allows the formation of hydrophilic gels of varying viscosity, which exhibit different dissolution kinetics of p-coumaric acid. In gels with higher viscosity, the amount of released *p*-coumaric acid decreased significantly. The obtained data confirm that CMC can prolong the release of the active substance [45]. From the 7% gel, a similar amount of *p*-coumaric acid was released as from the control capsule, indicating that this concentration of the gel does not modify the release of *p*-coumaric acid and, at the same time, lacks solubility-enhancing properties. CMC forms a clear viscous solution in water and can be used as a technological excipient (functional group: emulsifier, stabilizer, binder, thickener, and gelling agent) in pharmaceutical, food, veterinary, and cosmetic products [26,46].

Group C3 capsules also showed slow dissolution kinetics in the acidic medium, as illustrated in Figure 4e. Due to the presence of chitosan, these capsules released approximately three times less *p*-coumaric acid compared to the control group. The highest release in group C3 in acidic medium was observed in the C3-3 subgroup, but the value remained low at 15.57%. Although chitosan is soluble in diluted acids, its ability to form a hydrogel layer contributes to the prolonged release of the active compound from the dosage form. The dissolution profiles of group C3 capsules in phosphate buffer are presented in Figure 4f. Increasing chitosan concentration in the C3 capsule mixtures further slowed down dissolution kinetics, leading to very low release efficiency. A statistically significantly higher amount of *p*-coumaric acid was released from gel G3C compared to the capsules. This may be attributed to the fact that the gel structure was already formed, and the selected acceptor media had less influence on changes in the matrix interactions. A greater amount of *p*-coumaric acid was released in the buffer medium. It can be explained by the influence of the acidic environment on gel formation, hydrogen bonding and hydrophobic interactions can affect the matrix structure and thereby slow the release of the active compound—*p*-coumaric acid (Marinea et al., 2023) [37].

The dissolution of *p*-coumaric acid in PBS medium was evaluated using the Higuchi model. The dissolution curves of most capsule and gel formulations fitted this model well (R^2^ 0.78–0.94). This indicates that in these polymeric matrices, the release of *p*-coumaric acid is largely governed by diffusion, although additional mechanisms related to the use of swelling polymers (P407, NaCMC, chitosan)—such as matrix swelling, gel layer formation, or erosion—cannot be excluded.

The Higuchi constant (k) varied widely among the formulations, reflecting differences in the diffusion-controlled release rate. The highest k values were obtained for gels G1C and G2C and for capsules C1-2 and C2-3, indicating faster release of *p*-coumaric acid from these systems. Chitosan-containing capsules C3-1 and C3-2 showed the lowest k values and the poorest fit to the Higuchi model (R^2^ = 0.56–0.69), suggesting slower release, more strongly influenced by matrix swelling and structural changes [47]. In 0.1 M HCl, a similar release pattern was observed. The Higuchi model could also be considered an appropriate first approximation for describing release (R^2^ range 0.65–0.97), demonstrating clear differences in release rate between the various polymeric matrices. The fastest release was observed for P407-containing capsules C1-1 and C1-2 and gels G1C and G4C, whereas NaCMC and chitosan-based capsules (C2 and C3 series) exhibited the lowest k values and a weaker Higuchi fit, indicating slower and more complex release associated with matrix swelling. Comparing both media, the Higuchi model generally described the release process slightly better in PBS, where a strong correlation was observed between the solubility of *p*-coumaric acid and its antioxidant activity, underscoring the importance of physiologically relevant conditions when evaluating the performance of the formulated systems.

### 2.4. Antioxidant Activity

**Figure 5:**
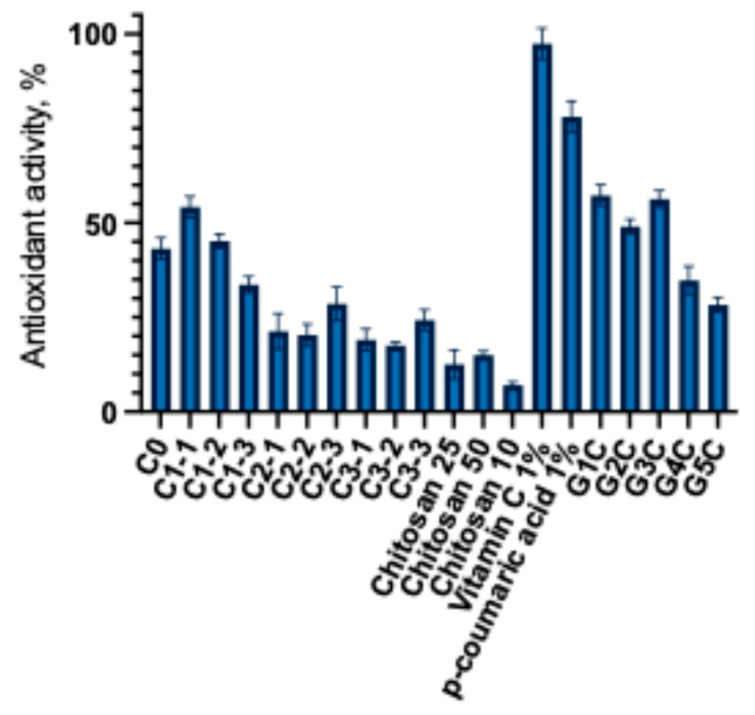
Antiradical activity of capsules and gels (mean ± SD, *n* = 3).

*p*-coumaric acid (4-hydroxycinnamic acid), a hydroxylated derivative of cinnamic acid, possesses antioxidant activity [48]. Various analytical methods have been used to evaluate the antioxidant properties of *p*-coumaric acid in vitro, including iron thiocyanate assay, DPPH, ABTS, and superoxide radical scavenging assays [49]. In this study, the antioxidant activity of the buffer acceptor media containing the amount of *p*-coumaric acid released from the gel matrix and capsules was determined using the DPPH method. The determined antiradical activity of the capsules and gels is presented in Figure 5.

This experimental study established that the antiradical activity depends on the amount of released *p*-coumaric acid and the presence of chitosan. The highest antioxidant activity was observed in formulations that released the largest amounts of *p*-coumaric acid. Capsules C1-1 and C1-2 showed the highest antiradical activity compared to the control. Capsules of the C2 and C3 groups showed lower antiradical activity. The C3 group capsules exhibited a similar antioxidant activity to that of the C2 group capsules. Although a lower amount of p-coumaric acid was released from the C3 group capsules, as shown in the data presented in Figure 5, the addition of chitosan may have influenced the antioxidant activity of the C3 group capsules. Analysis of the modeled gels revealed that formulations G1C and G3C exhibited the strongest antiradical activity. The results indicate that the chitosan-based gel itself possesses intrinsic antioxidant properties, which enhanced the overall radical scavenging capacity of the formulations. Other researchers have also employed the DPPH radical scavenging method to characterize the antioxidant activity of p-coumaric acid and found that its DPPH radical scavenging activity increased in a dose-dependent manner [4,50]. After evaluating the antioxidant activity of the modeled capsules and gels, a statistically significant difference was found compared with the control 1% ascorbic acid and *p*-coumaric acid solutions. This further confirms that excipients influence the release of active compounds and their antioxidant activity. Additionally, Chinese researchers successfully incorporated *p*-coumaric acid into multifunctional chitosan coatings that demonstrated strong radical scavenging effects [51]. Carboxymethyl cellulose (CMC) is widely used in biomedical, pharmaceutical, textile, and cosmetic industries [52,53]. The results of this study highlight the potential application of this polymer in the development of *p*-coumaric acid-based gels. Furthermore, the functionality of CMC can be enhanced by designing extended-release formulations containing *p*-coumaric acid. To overcome the mentioned limitations, current research trends are focused on the chemical modification of CMC, the development of composite formulations, and the incorporation of bioactive compounds that improve its performance and functionality [54]. The preliminary results of our DPPH radical scavenging experiments confirm the relevance of further investigating the interactions between *p*-coumaric acid and polymeric materials, as well as their underlying mechanisms of activity. The relationship between dissolution and antioxidant activity was evaluated using Pearson correlation. In 0.1 M HCl medium no statistically significant correlation was found between antioxidant activity and dissolution (*r* = −0.21, R^2^ = 0.04, *p* = 0.47). In PBS medium a very strong positive correlation was observed (*r* = 0.91, R^2^ = 0.83, *p* < 0.0001), indicating that as the amount of *p*-coumaric acid released in PBS increases its antioxidant activity increases. These results show that improving dissolution in acidic 0.1 M HCl medium does not have a clear relationship with the antioxidant activity of *p*-coumaric acid, in PBS medium the relationship between dissolution and antioxidant activity is very strong. Formulations in which the polymer system ensures greater dissolution of *p*-coumaric acid in PBS also exhibit higher antioxidant activity. This confirms that the differing compositions of capsules and gels (poloxamer, NaCMC, and chitosan) determine the release behavior of the active compound and directly influence its antioxidant potential.

## 3. Conclusions

The selected polymers modified the solubility of *p*-coumaric acid. The dissolution kinetics of *p*-coumaric acid depended on the type and amount of polymeric material as well as on the pharmaceutical dosage form. The release of the active substance from the modeled formulations was governed by the interactions between the drug, the polymers, and the acceptor medium. The strongest antioxidant activity was observed in those formulations that released the highest amount of *p*-coumaric acid, with chitosan also contributing to the overall antioxidant effect. The results of this study confirm that the type and concentration of excipients significantly affect the quality of capsule and gel formulations. *p*-coumaric acid shows potential for use as a natural antioxidant. Further studies are required to evaluate its safety and efficacy and to develop optimized food-supplement compositions containing *p*-coumaric acid. Future research should focus on determining scientifically justified optimal concentrations of poloxamers and exploring innovative technological approaches to enhance the solubility of *p*-coumaric acid. Compositions of *p*-coumaric acid with polymers may be applied both in the food and pharmaceutical fields, as well as in the development of biologically active formulations with beneficial effects on human health. Increasing the concentration of polymeric excipients resulted in slower *p*-coumaric acid kinetics; however, it is appropriate to examine the influence of lower concentration polymers on the biopharmaceutical properties of the formulation. Nevertheless, these valuable compositions need to be investigated in greater detail in order to elucidate and understand the interactions between the polymer and the active substance and their impact on physicochemical properties and biological activity.

## 4. Materials and Methods

### 4.1. Materials

Manual capsule filling machine (Capsuline, Fort Lauderdale, FL, USA), thermostatic shaker (GFL, Burgwedel, Germany), disintegration tester AT7 Smart (SOTAX, Aesch, Switzerland), analytical balance Scaltec SBC 31 (Scaltec Instruments GmbH, Göttingen, Germany), magnetic stirrer with heating plate IKA C-MAG HS 7 (IKA-Werke GmbH & Co. KG, Staufen im Breisgau, Germany), UV/Vis spectrophotometer Agilent 8453 (Agilent Technologies, Inc., Santa Clara, CA, USA). *p*-coumaric acid (≥98%; Sigma–Aldrich, St. Louis, MO, USA), silicified microcrystalline cellulose Prosolv SMCC 50 (JRS Pharma, Rosenberg, Germany), poloxamer 407 (Kolliphor P407, BASF Pharma, Ludwigshafen, Germany), sodium carboxymethyl cellulose (medium viscosity; Sigma-Aldrich, St. Louis, MO, USA), chitosan (medium molecular weight; Sigma-Aldrich, St. Louis, MO, USA), hydrochloric acid solution (≥99.8%; Sigma–Aldrich, St. Louis, MO, USA), phosphate buffer tablets (pH 6.8; Gibco, Thermo Fisher Scientific, Paisley, UK), rectified food-grade ethanol (96% *v*/*v*; Vilniaus degtinė, Vilnius, Lithuania), DPPH (2,2-diphenyl-1-picrylhydrazyl radical) reagent (Sigma-Aldrich, St. Louis, MO, USA), Folin–Ciocalteu reagent (2 M; Sigma–Aldrich, Buchs, Switzerland), sodium carbonate (Sigma-Aldrich, St. Louis, MO, USA).

### 4.2. Formulation and Production of Capsules Hydrogels Containing p-Coumaric Acid

Capsules were prepared using size 0 hard gelatin shells. The encapsulated content consisted of powdered mixtures obtained by thoroughly blending *p*-coumaric acid with selected excipients (Table 2a). Various capsule formulations were assembled with a capsule-filling device (Capsuline, Davie, FL, USA). Experimental groups of capsules containing *p*-coumaric acid were designed. Silicified microcrystalline cellulose (PROSOLV SMCCTM 50) was selected as the capsule filler due to its ability to enhance solubility. For the compositions of capsule groups C1-C3, it was decided to additionally incorporate excipients with properties to modify release, improve flowability, or promote disintegration—poloxamer 407, sodium carboxymethyl cellulose, and chitosan. Powder mixtures were filled into size 0 hard gelatin capsules. The prepared capsules were stored in sealed plastic containers, in a dark place at room temperature.

Hydrogels containing *p*-coumaric acid were prepared on a weight-by-weight (*w*/*w*) basis according to the compositions presented in Table 2b. For the experimental work, polymer concentrations were selected that produced semi-solid systems and closely matched the polymer fraction used in the modeled capsule formulations. Poloxamer 407 gels were prepared when required amount of polymer was dissolved in purified water and kept at 4 °C for 48 h until a clear solution was obtained. Chitosan gels were prepared by dissolving chitosan powder in water under continuous stirring on a hot plate, followed by the gradual addition of 30% acetic acid until complete dissolution and gel formation. NaCMC hydrogels were obtained by dispersing the polymer in purified water at room temperature under gentle stirring until a clear solution was formed. All formulations were adjusted to a total mass of 100 g (*w*/*w*) and stored at 4 °C until further analysis. The suspended form of *p*-coumaric acid was incorporated into the prepared bases.

### 4.3. Quantitative Analysis of p-Coumaric Acid

The quantitative analysis of *p*-coumaric acid was performed using spectrophotometric methods, adapted with certain modifications from the procedure described by Panda et al. [55]. For the analysis, 0.4 mL of the *p*-coumaric acid sample solution was transferred into test tubes, followed by the addition of 1 mL of Folin–Ciocalteu reagent. Subsequently, 3 mL of a 2% sodium carbonate solution was added. The mixtures were thoroughly mixed and left to stand in a dark environment for 2 h, with occasional mixing during this period. After incubation, the contents of the test tubes were filtered and analyzed using a UV/Vis spectrophotometer at a wavelength of 760 nm. The obtained results were expressed as percentages.

### 4.4. p-Coumaric Acid Solubility

The solubility of *p*-coumaric acid was evaluated in different media, including HCl + NaCl buffer (pH 1.2), phosphate buffer (pH 6.8), purified water and 96% ethanol following the method described by Hussain et al. [56]. An excess amount of *p*-coumaric acid (500 mg) was added to 50 mL of medium in a conical flask, sealed, and placed in a thermostatic shaker at 25 °C, 100 rpm, for 24 h to reach equilibrium. After incubation, the suspensions were filtered through a 0.45 µm syringe filter. The filtrate was analyzed using UV-Vis spectrophotometry.

### 4.5. Evaluation of the Physicochemical Properties of Gel Bases

The viscosity of the gels was determined using a rotational viscometer (Fungilab, Barcelona, Spain) at controlled temperatures of 22 °C and 37 °C, with the instrument set to operate at a speed of 20 rpm. The pH values of the gels were measured at 23 ± 1 °C using a Knick 766 Calimatic pH meter (Knick Elektronische Messgeräte GmbH & Co. KG, Berlin, Germany).

### 4.6. In Vitro Disintegration Test of Capsules

The disintegration time of the capsules was determined using the magnetic stirring method. Analyzing the disintegration kinetics of capsules, a laboratory beaker containing 50 mL of purified water was placed on a magnetic stirrer with a stirring bar, and the medium was heated to 37 ± 0.5 °C while maintaining a stirring speed of 700 rpm. A capsule of the corresponding formulation was then introduced into the beaker, and the starting time was recorded. The disintegration endpoint was defined as the moment when the capsule had completely disintegrated into a uniform soft mass. To ensure accuracy and reproducibility, the test was performed using six capsules. If at least one of these capsules failed to disintegrate within the time specified by the European Pharmacopoeia, an additional twelve capsules were tested [57]. Out of the total eighteen capsules examined, at least sixteen were required to disintegrate within the specified time limit to meet the acceptance criteria.

### 4.7. In Vitro Dissolution Test of Capsules and Gels

The dissolution test for capsules was conducted in accordance with the methodology described in the European Pharmacopoeia for solid dosage forms (Ph. Eur. 2.9.3) [58]. Analyzing the dissolution kinetics of capsules, a paddle apparatus with a sinker, which operates based on horizontal rotation of paddles and continuous mixing of the medium, was used for the test. For the investigation of *p*-coumaric acid dissolution kinetics from gels, a paddle apparatus was also used, with 5.0 g of the test gel added to the dissolution medium. A volume of 500 mL of the appropriate dissolution medium—purified water, hydrochloric acid with sodium chloride (pH 1.2), or phosphate-buffer solution (pH 6.8)—was placed into the apparatus vessel. The vessels were continuously stirred with the paddle device and maintained at a temperature of 37 ± 0.5 °C. Once the medium reached the required temperature, a capsule was introduced into the vessel, and the start time of the test was recorded. At predetermined time intervals (5, 10, 15, 30, 45, 60, and 90 min), 5 mL samples were withdrawn from the dissolution medium. To maintain a constant volume, an equal amount of fresh dissolution medium was added after each sampling. The collected samples were prepared for analysis by filtering 1 mL of each sample and diluting it to a final volume of 10 mL with the respective dissolution medium. Further procedures are described in Methods, Section 2.3.

### 4.8. Evaluation of Antioxidant Activity

The test was performed following the methodology described by Rezzoug et al. [59]. A 60 µM DPPH solution was prepared by dissolving the appropriate amount of reagent in 96% (*v*/*v*) ethanol. Samples were obtained either (a) from PBS dissolution media at predefined time points and filtered (0.45 µm) or (b) by dispersing a known gel mass in ethanol:buffer (1:1, *v*/*v*) and filtering (0.45 µm). 1 mL of sample was mixed with 2 mL of DPPH solution. The reaction mixtures were allowed to stand for 30 min, protected from light to maintain DPPH stability throughout the reaction period. At the end of the reaction, the absorbance of the 60 µM DPPH solution and the reacted samples was measured using a UV/Vis spectrophotometer at a wavelength of 517 nm. The antioxidant activity of *p*-coumaric acid was calculated and expressed as a percentage using the designated formula [60].

Antiradical activity = (A1 − A2)/A1 · 100%

where A1—60 µM absorbance of the DPPH solution, and A2—absorbance of the DPPH solution mixed with the sample after the reaction [60].

### 4.9. Statistical Analysis

Statistical analysis of the experimental data was performed using GraphPad Prism 10 (version 10.0.2, GraphPad Software, LLC, San Diego, CA, USA; 2023). The results were expressed as mean from three measurements (n = 3), standard deviations, and standard errors. One-way analysis of variance (ANOVA) was used to assess differences between subgroups, followed by Tukey’s multiple comparison test as the post hoc analysis. Differences were considered statistically significant when the *p*-value was less than 0.05 (*p* < 0.05). Drug release profiles were analyzed using the Higuchi model (Q_t_ = k·t^1/2^) by linear regression of the cumulative amount released versus the square root of time. The relationship between dissolution and antioxidant activity was evaluated using Pearson correlation analysis.

## Figures and Tables

**Table 1:** Physicochemical properties of prepared gels G1C-G5C at day 1 and after 30 days (mean ± SD, n = 3).

No	pH (Mean ± SD)	pH (Mean ± SD) After 30 Days	Viscosity 22 °C (mPa·s)	Viscosity 22 °C (mPa·s) After 30 Days	Viscosity 37 °C (mPa·s)	Viscosity 37 °C (mPa·s) After 30 Days	Appearance
G1C	5.05 ± 0.25	5.19 ± 0.14	43.1 ± 2.2	37.9 ± 2.0	320 ± 16	299.7 ± 9.0	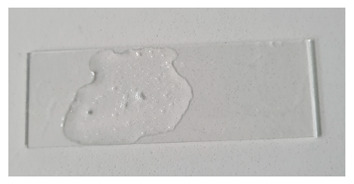
G2C	5.50 ± 0.28	5.55 ± 0.16	5500 ± 275	5470.3 ± 45.1	4770 ± 239	4698.0 ± 190	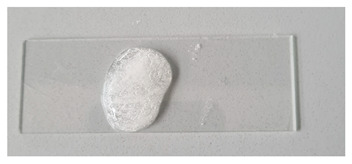
G3C	3.71 ± 0.19	3.51 ± 0.17	8029 ± 401	80,213 ± 293	7487 ± 374	7604 ± 236	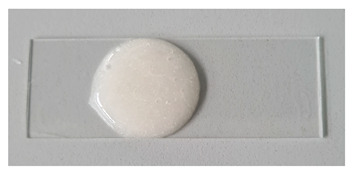
G4C	5.57 ± 0.28	5.64 ± 0.12	26,271 ± 1314	26,459.3 ± 525	58,255 ± 2913	57,971 ± 952	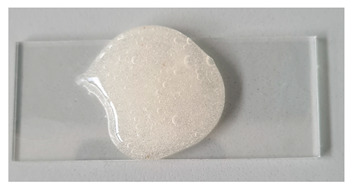
G5C	5.81 ± 0.29	5.83 ± 0.13	48,773 ± 2439	49,141 ± 907	32,465 ± 1623	32,238 ± 564	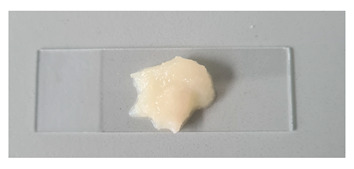

**Table 2:** Compositions of the formulated capsules (**a**) and gels (**b**) containing *p*-coumaric acid.

(a)							
**Group**	*p*-Coumaric Acid Content, mg	PROSOLV SMCCTM 50 mg	P407, mg	NaCMC, mg	Chitosan, mg	Polymer Concentration in Capsule	Mean Mass of Capsule, mg (mean ± SD)
C0	100	-	-	-	-		99.7 ± 2.3
C1-1		50	25	-	-	14.3%	174.0 ± 2.0
C1-2		50	50	-	-	25%	198.7 ± 2.1
C1-3		50	100	-	-	40%	252.7 ± 5.9
C2-1		50	-	25	-	14.3%	175.7 ± 2.3
C2-2		50	-	50	-	25%	199.0 ± 3.6
C2-3		50	-	10	-	6.67%	153.3 ± 1.5
C3-1		50	-	-	25	14.3%	175.3 ± 2.3
C3-2		50	-	-	50	25%	201.0 ± 2.6
C3-3		50	-	-	10	6.67%	159.7 ± 2.3
(**b**)							
**Group**	***p*-Coumaric Acid Content, g**	**P407, g**		**NaCMC, g**	**Chitosan, g**		**Water**
G1C	1	14					Add 100
G2C				7			
G3C					5		
G4C		25					
G5C				14			

## Data Availability

Data is unavailable due to privacy or ethical restrictions.
